# The zebrafish *merovingian* mutant reveals a role for pH regulation in hair cell toxicity and function

**DOI:** 10.1242/dmm.016576

**Published:** 2014-07

**Authors:** Tamara M. Stawicki, Kelly N. Owens, Tor Linbo, Katherine E. Reinhart, Edwin W. Rubel, David W. Raible

**Affiliations:** 1 Department of Biological Structure, University of Washington, Seattle, WA 98195, USA.; 2 Virginia Merrill Bloedel Hearing Research Center, University of Washington, Seattle, WA 98195, USA.; 3 Department of Otolaryngology, Head and Neck Surgery, University of Washington, Seattle, WA 98195, USA.

**Keywords:** Aminoglycosides, Cisplatin, Hair cells, H^+^-ATPase, Ototoxicity, pH

## Abstract

Control of the extracellular environment of inner ear hair cells by ionic transporters is crucial for hair cell function. In addition to inner ear hair cells, aquatic vertebrates have hair cells on the surface of their body in the lateral line system. The ionic environment of these cells also appears to be regulated, although the mechanisms of this regulation are less understood than those of the mammalian inner ear. We identified the *merovingian* mutant through genetic screening in zebrafish for genes involved in drug-induced hair cell death. Mutants show complete resistance to neomycin-induced hair cell death and partial resistance to cisplatin-induced hair cell death. This resistance is probably due to impaired drug uptake as a result of reduced mechanotransduction ability, suggesting that the mutants have defects in hair cell function independent of drug treatment. Through genetic mapping we found that *merovingian* mutants contain a mutation in the transcription factor *gcm2*. This gene is important for the production of ionocytes, which are cells crucial for whole body pH regulation in fish. We found that *merovingian* mutants showed an acidified extracellular environment in the vicinity of both inner ear and lateral line hair cells. We believe that this acidified extracellular environment is responsible for the defects seen in hair cells of *merovingian* mutants, and that these mutants would serve as a valuable model for further study of the role of pH in hair cell function.

## INTRODUCTION

Hearing loss is currently the most prevalent sensory disorder; about 10% of adults and 35% of people over 65 suffer from hearing impairment (Davis, 1989; Ries, 1994). The inner ear is highly sensitive to damage, and numerous genetic mutations and environmental insults lead to hearing loss (Dror and Avraham, 2009; Rybak and Ramkumar, 2007; Sliwinska-Kowalska and Davis, 2012). The inner ear is enriched in ionic transporters also highly expressed in the kidney, such as the H^+^-ATPases and Cl^−^/HCO_3_^−^ exchangers (Lang et al., 2007), suggesting a role for ionic homeostasis in the functioning of the audiovestibular system. Active pH regulation in the inner ear is suggested by studies showing altered pH of endolymph and the endolymphatic sac following treatment with carbonic anhydrase or H^+^-ATPase inhibitors (Couloigner et al., 2000; Sterkers et al., 1984). Additionally, mutations in H^+^-ATPase transporter subunits cause hearing loss in the human disease distal rental tubular acidosis (dRTA) and in mouse models of this disease (Hennings et al., 2012; Karet et al., 1999; Norgett et al., 2012; Smith et al., 2000).

Aquatic vertebrates also control the ionic environment of hair cells of the lateral line system. Lateral line hair cells are located on the surface of the animal, with apical structures protruding into the water enclosed in a gelatinous matrix called the cupula. The ionic environment of the cupula differs from the surrounding water, suggesting active ionic regulation (McGlone et al., 1979; Russell and Sellick, 1976). However, the mechanisms of this regulation are not known. Ionic homeostasis is a particular challenge for freshwater fish, due to ion loss by diffusion into their environment (Dymowska et al., 2012). To combat this problem, fish use specialized cells enriched in ionic transporters called ionocytes (Evans et al., 2005; Hwang and Lee, 2007). It is believed that the gills and the associated ionocytes are the primary site of osmoregulation in fish rather than the kidneys (Evans et al., 2005). One type of ionocyte, the H^+^-ATPase-rich ionocyte, expresses high levels of the H^+^-ATPase transporter and the Cl^−^/HCO_3_^−^ exchanger SLC4A1B, and contributes to pH regulation (Lee et al., 2011; Lin et al., 2006).

Hair cells of the lateral line are susceptible to the same ototoxic drugs as mammalian inner ear hair cells, including aminoglycoside antibiotics and chemotherapeutics (Harris et al., 2003; Ou et al., 2007; Ton and Parng, 2005). We have used the zebrafish lateral line system to screen for genes involved in aminoglycoside toxicity (Owens et al., 2008). In this report we show that that the *merovingian* (*mero*) mutant is resistant to both neomycin- and cisplatin-induced hair cell death due to impaired uptake of these toxicants into hair cells. The gene responsible for the defects in *merovingian* mutants is *gcm2*, a transcription factor important for the generation of H^+^-ATPase-rich ionocytes (Chang et al., 2009). We show that *merovingian* mutants have an acidified extracellular environment in the vicinity of hair cells of both the lateral line and inner ear. Thus, the *merovingian* mutant and zebrafish lateral line might be useful model systems to assess the role of pH regulation in hair cell function.

## RESULTS

### *merovingian* mutants are resistant to multiple hair cell toxicants

**Fig. 1.**
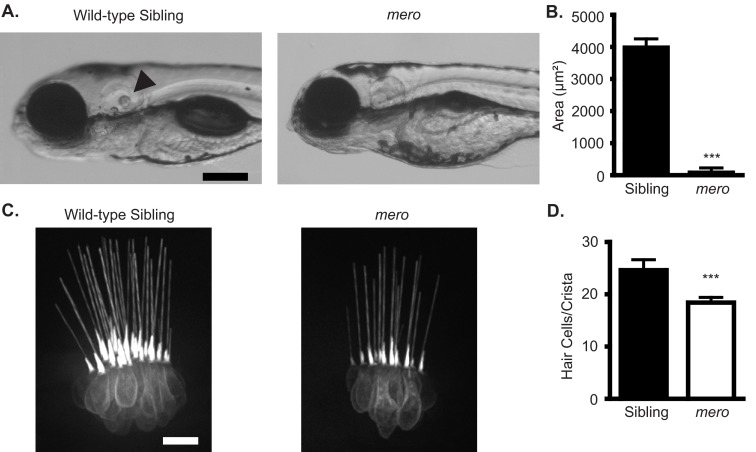
***merovingian* mutants have inner ear defects.** (A) *merovingian* (*mero*) mutants show multiple phenotypes including a failure to inflate their swim bladders, an enlarged yolk, and impaired otolith formation. Arrowhead points to otolith. (B) Quantification of the size of the posterior otolith in wild-type siblings and *merovingian* mutants. Otolith size is significantly reduced in *merovingian* mutants. Mutants were selected randomly and included eight fish lacking a posterior otolith and therefore having an otolith size of 0 (*n*=10 fish). (C) Hair cells expressing the *brn3c:gfp* transgene in the lateral crista of both wild-type siblings and *merovingian* mutants. (D) Quantification of the number of hair cells/crista in wild-type siblings and *merovingian* mutants. All three crista were used for counting. There is a significant reduction in hair cell number in *merovingian* mutants (*n*=9 fish). ***P<0.0001 by Student’s *t*-test; error bars indicate s.d. Scale bars: 250 μm (A), 10 μm (C).

The *merovingian* mutant was identified in a genetic screen for mutations that conferred resistance to neomycin-induced hair cell death (Owens et al., 2008). *merovingian* mutants show a number of phenotypes in addition to resistance to neomycin-induced hair cell death, including a failure to inflate their swim bladders, an enlarged yolk, and impaired otolith formation (Fig. 1A,B). The average size of the posterior otolith in wild-type zebrafish larvae at 5 days post-fertilization (dpf) was 3,970±275 μm^2^, whereas in *merovingian* mutants the average size of this otolith was reduced to 71±155 μm^2^, with otoliths being absent in the majority of mutants. Additionally, *merovingian* mutants show behavioral defects, commonly seen in zebrafish mutants with impaired hair cell mechanotransduction, including an inability to remain upright, failure to respond to acoustic/vibrational stimuli (tapping on the dish) (supplementary material Movie 1) and circling behavior (supplementary material Movie 2). These phenotypes allow the separation of *merovingian* mutants from their wild-type siblings and, therefore, a further characterization of their defects in hair cell development, function and response to toxicants.

**TRANSLATIONAL IMPACT**

**Clinical issue**

Hearing loss affects about 10% of the adult human population. The inner ear hair cells, which detect sound and transmit it to the brain, are highly sensitive to damage, and numerous genetic mutations and environmental insults, particularly exposure to ototoxic drugs such as aminoglycoside antibiotics and chemotherapeutics, can lead to hearing loss. The degree of hearing loss in response to ototoxic medications varies greatly from patient to patient. This variability is thought to be partly due to genetic differences. Like mammals, zebrafish have inner ear hair cells but, in addition, they have a lateral line system consisting of hair cells on the surface of their body that detect water motion. Lateral line hair cells are responsive to the same ototoxins as mammalian inner ear hair cells. Consequently, the zebrafish lateral line system can be used in unbiased genetic screens to identify novel genes involved in general hair cell function and in hair cell responses to ototoxic drugs.

**Results**

Here, the authors use the zebrafish lateral line system to screen for genes involved in neomycin- and cisplatin-induced toxicity. They identify a mutation in the transcription factor gene *gcm2* that makes the lateral line hair cells resistant to both drugs. This resistance appears to be due to impaired mechanotransduction ability as the *merovingian* mutants also show audiovestibular behavioral defects. *gcm2* is important for the production of ionocytes, cells that are crucial for whole body pH regulation in fish, and the *merovingian* mutants show acidification of the extracellular environment throughout their body. Notably, the extracellular but not the intracellular environment of the lateral line hair cells in the mutants is acidified, which suggests that changes in extracellular pH are responsible for the defects seen in these mutants.

**Implications and future directions**

This work provides the second example of a gene that is important for pH regulation that affects the response of hair cells to ototoxic drugs and suggests that pH regulation has a key role in this process. This study and conclusion are supported by the presence of sensorineural hearing loss in distal renal tubular acidosis, a disorder that is caused by mutations in the pH-regulating H^+^-ATPase complex, they also support a role for pH regulation in normal hair cell function. Because the extracellular environment around the hair cells in *merovingian* mutants is acidified, these mutants can now be used as a model system in which to study the role of pH regulation in the function of hair cells and their response to ototoxic drugs.

**Fig. 2.**
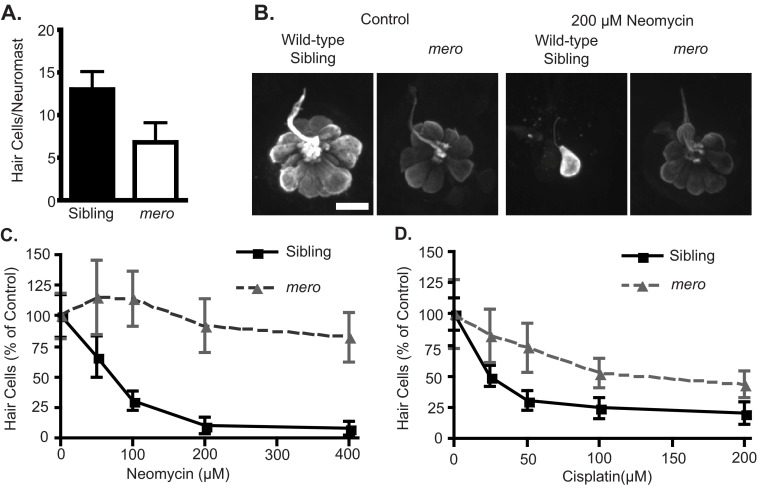
***merovingian* mutants are resistant to toxicant-induced hair cell death.** (A) Quantification of lateral line hair cell number in wild-type siblings and *merovingian* mutants; ***P<0.0001 by Student’s *t*-test (*n*=10 fish). (B) Hair cells labeled with parvalbumin in *merovingian* mutants and wild-type siblings without (left) and with (right) neomycin treatment. *merovingian* mutants show reduced initial hair cell numbers, but no hair cell loss in response to neomycin. (C) *merovingian* mutants show a significant resistance to neomycin-induced hair cell death; *P*<0.0001 by two-way ANOVA (*n*=10 fish). (D) *merovingian* mutants are partially resistant to cisplatin-induced hair cell death. Genotypes are significantly different; *P*<0.0001 by two-way ANOVA (*n*=6–10 fish). Error bars indicate s.d. Scale bar: 10 μm.

Fish expressing a membrane-targeted GFP under the control of the *brn3c(pou4f3)* promoter (*brn3c:gfp*) (Xiao et al., 2005) were used to label hair cells of the inner ear. We found that the inner ear hair cells of *merovingian* mutants show grossly normal morphology; however, there was a slight reduction in total hair cell number, with wild-type siblings averaging 24.7±1.9 hair cells/crista and *merovingian* mutants averaging 18.3±1.0 hair cells/crista (Fig. 1C,D). To look at lateral line hair cells, we labeled them with an anti-parvalbumin antibody (Hsiao et al., 2002; Steyger et al., 1997) and counted the hair cells in six specific neuromasts of the anterior lateral line (see Materials and Methods). *merovingian* mutants showed a significant reduction in lateral line hair cell number as compared with their wild-type siblings, with wild-type siblings averaging 13.0±2.1 hair cells/neuromast and *merovingian* mutants averaging 6.8±3.4 hair cells/neuromast (Fig. 2A,B). Levels of parvalbumin staining in *merovingian* mutants were reduced compared with wild-type siblings, although hair cell morphology appeared otherwise normal (Fig. 2B). Using anti-parvalbumin labeling, we examined the effect of two classes of hair cell toxicants on hair cells in *merovingian* mutants. We found that *merovingian* mutants show significant resistance to the aminoglycoside antibiotic neomycin across a concentration range of 50–400 μM (Fig. 2B,C) and a partial resistance to the chemotherapeutic cisplatin across a concentration range of 25–200 μM (Fig. 2D). The small increases in average hair cell numbers of *merovingian* mutants treated with 50 and 100 μM neomycin as compared with control fish only exposed to embryo media (EM) (Fig. 2C) are not significant.

### *merovingian* mutants show impaired uptake of FM1-43 and hair cell toxicants

Uptake of both aminoglycoside antibiotics and cisplatin into hair cells of the zebrafish lateral line is dependent upon functional mechanotransduction (Alharazneh et al., 2011; Gale et al., 2001; Marcotti et al., 2005; Thomas et al., 2013). As *merovingian* mutants are resistant to both these toxicants and show vestibular defects, we hypothesized that resistance to hair cell toxicants might result from reduced drug uptake due to impaired mechanotransduction.

**Fig. 3.**
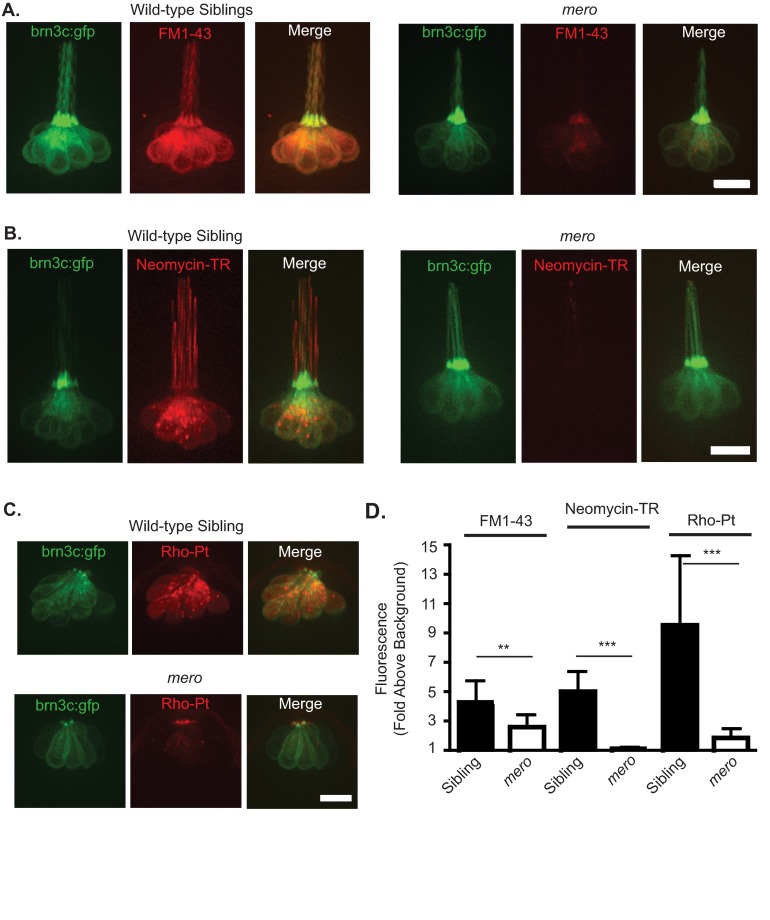
**Uptake of FM1-43 and hair cell toxicants are reduced in *merovingian* mutants.** (A) There is reduced uptake of FM1-43 into the hair cells of *merovingian* mutants. Hair cells are expressing the *brn3c:gfp* transgene. (B) Neomycin-TR fails to enter hair cells of *merovingian* mutants. (C) Rho-Pt uptake is reduced in *merovingian* mutants. (D) Quantification of FM1-43, neomycin-TR, and Rho-Pt uptake into hair cells, normalized to background fluorescence (*n*=10 fish); **P=0.0046, ***P<0.0001 for neomycin-TR and *P*=0.0006 for Rho-Pt by Student’s *t*-test, Welch’s correction was used for neomycin-TR and Rho-Pt. Error bars indicate s.d. Scale bars: 10 μm.

To investigate mechanotransduction in *merovingian* mutants we used the vital dye FM1-43, in which rapid uptake (≤1 minute) is mechanotransduction-dependent (Gale et al., 2001; Meyers et al., 2003; Seiler and Nicolson, 1999). Fish expressing the *brn3c:gfp* transgene were used to allow visualization of hair cells. These fish were exposed to FM1-43 for 1 minute and then imaged. *merovingian* mutants showed a significant reduction in FM1-43 uptake, with the fluorescent intensity/background measurement of FM1-43 being 4.3±1.5 in wild-type siblings as compared with 2.6±0.9 in *merovingian* mutants. This decrease in rapid FM1-43 loading is consistent with the hypothesis that mechanotransduction activity is decreased in these fish (Fig. 3A,D).

We next examined uptake of labeled versions of the toxicants neomycin and cisplatin. For neomycin uptake studies, we used neomycin covalently labeled with the fluorophore Texas Red (neomycin-TR). Fish were treated with 50 μM neomycin-TR for 15 minutes and then imaged. We found no significant entry of neomycin-TR into the hair cells of *merovingian* mutants, with the fluorescent intensity/background measurement of neomycin-TR being 1.1±0.1 in *merovingian* mutants as compared with 5.0±1.3 in wild-type siblings (Fig. 3B,D). This is consistent with the strong resistance of these mutants to neomycin-induced hair cell toxicity (Fig. 2B,C). To investigate cisplatin uptake, we used a rhodamine-conjugated platinum reagent (Rho-Pt) in which a cisplatin-like moiety is linked to the rhodamine derivative 6-TAMRA (Alers et al., 1999; van Gijlswijk et al., 2001). Rho-Pt has previously been used in zebrafish to investigate cisplatin uptake (Thomas et al., 2013). Fish were exposed to 25 μM Rho-Pt for 20 minutes and imaged. Rho-Pt entered hair cells in *merovingian* mutants, although its entry was significantly reduced, with the fluorescent intensity/background measurement of Rho-Pt being 9.6±4.7 in wild-type siblings as compared with 1.9±0.6 in *merovingian* mutants (Fig. 3C,D). This result is consistent with the partial resistance *merovingian* mutants show against cisplatin-induced hair cell loss (Fig. 2D).

### *merovingian* mutants contain a missense mutation in the transcription factor *gcm2*

**Fig. 4.**
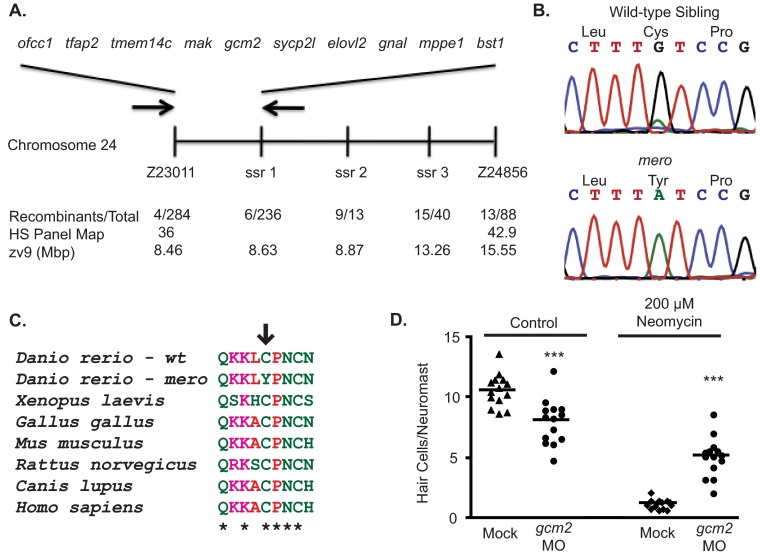
***merovingian* is a mutation in *gcm2*.** (A) *merovingian* was mapped to a ~170,000 bp region on chromosome 24 (arrows) containing 10 genes. Neighboring microsatellite markers used for mapping are shown as well as the number of recombinant animals found for each marker. (B) *gcm2* cDNA sequencing results from pooled groups of *merovingian* wild-type siblings and mutants. Mutants contain a G-to-A mutation resulting in a cysteine to tyrosine amino acid change. (C) The cysteine residue mutated in *merovingian* is conserved across numerous species. (D) Injection of a *gcm2* morpholino (MO) reduced hair cell number in control fish and causes neomycin resistance; ***P<0.001 by two-way ANOVA and Bonferroni post-hoc test (*n*=14 fish).

To identify the gene mutated in *merovingian* mutants, we performed genetic mapping using zebrafish microsatellite markers (Knapik et al., 1998; Shimoda et al., 1999). The *merovingian* mutation co-segregated with a region on chromosome 24 containing 10 genes (Fig. 4A), which were sequenced to identify potential mutations. Only one gene, *gcm2*, contained a coding sequence mutation. This G-to-A nucleotide change causes a cysteine to tyrosine amino acid change (Fig. 4B). This cysteine is highly conserved among diverse species (Fig. 4C).

*merovingian* mutants show many of the phenotypes previously reported in fish injected with *gcm2* antisense morpholino oligonucleotides (MO), including a failure to inflate their swim bladders, an enlarged yolk and impaired otolith formation (Fig. 1A) (Hogan et al., 2004). To test whether knockdown of *gcm2* would cause similar hair cell defects as seen in *merovingian* mutants, we injected fish with a *gcm2* MO. Like *merovingian* mutants, *gcm2* morphants showed a reduction in initial lateral line hair cell number(8.1±1.9 hair cells/neuromast as compared with 10.7±1.4 in controls) and resistance to neomycin-induced hair cell death (5.2±1.6 hair cells/neuromast following 200 μM neomycin as compared with 1.2±0.4 in controls) (Fig. 4D).

**Fig. 5.**
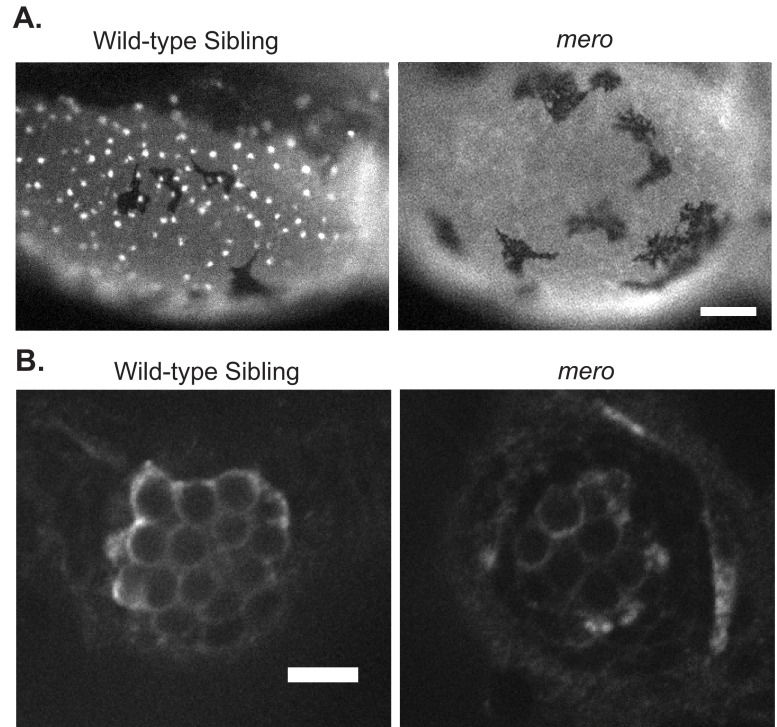
***merovingian* mutants lack H^+^-ATPase-rich ionocytes, but not hair cell H^+^-ATPases.** (A) vH-ATPase labeling on the yolk of 3-dpf zebrafish. *merovingian* mutants lack H^+^-ATPase-rich ionocytes present in wild-type siblings.(B) vH-ATPase labeling in neuromasts of 5-dpf zebrafish. Although reduced in level, staining is still present in *merovingian* mutants. Scale bars: 100 μm (A), 10 μm (B).

*gcm2* has previously been shown to be necessary for the production of H^+^-ATPase-rich ionocytes (Chang et al., 2009; Esaki et al., 2009). To confirm that *gcm2* function was impaired in *merovingian* mutants, we labeled H^+^-ATPase-rich ionocytes in 3-dpf zebrafish larvae by staining with an anti-vH-ATPase antibody. Robust staining was present on the yolk of wild-type zebrafish larvae and was absent in *merovingian* mutants (Fig. 5A). We also observed an enrichment of H^+^-ATPase staining in the vicinity of lateral line hair cells (Fig. 5B). This is in agreement with previous reports showing that H^+^-ATPases are expressed in hair cells (Shiao et al., 2005; Stanković et al., 1997). This staining, although reduced in level, was still present in *merovingian* mutants (Fig. 5B).

### *merovingian* mutants show a whole body acidification, including in the extracellular environment of hair cells

*gcm2* expression in zebrafish is believed to be restricted to the pharyngeal arches and ionocytes and is not expressed in hair cells or support cells (Chang et al., 2009; Hanaoka et al., 2004; Hogan et al., 2004; Shono et al., 2011). This suggests that *gcm2* acts globally to influence hair cells. *gcm2* morphants have been shown to have impaired whole body proton excretion (Chang et al., 2009). We hypothesized that this impaired proton excretion would lead to internal acidification of the animal and, in turn, influence hair cell function. To test for acidification, we used the ratiometric pH-sensitive fluorescent protein pHluorin2 (Mahon, 2011). Ratiometric pHluorin contains two excitation peaks, one at 395 nm and one at 475 nm. The fluorescence intensity for the excitation peak at 395 nm decreases with decreasing pH, whereas that for the excitation peak at 475 nm increases with decreasing pH (Miesenböck et al., 1998).

**Fig. 6.**
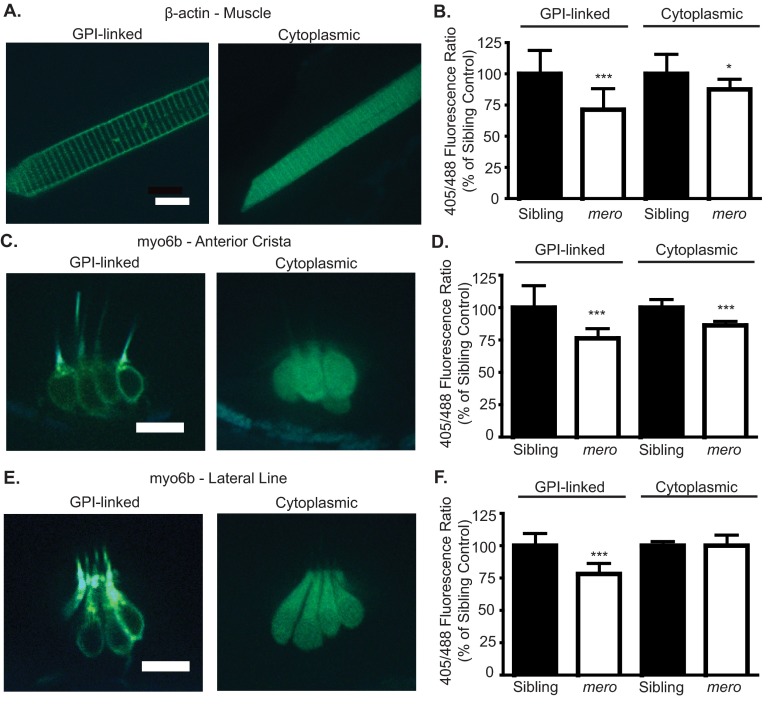
***merovingian* mutants show a decreased in extracellular pH throughout their body.** (A) Images of both GPI-linked and cytoplasmic pHluorin2 expressed under the control of the *β-actin* promoter in muscle cells. (B) The extracellular (GPI-linked) and intracellular (cytoplasmic) environment of muscle cells is acidified in *merovingian* mutants, as measured by a decreased 405/488 ratio in the ratiometric fluorescent pH indicator pHluorin2 (*n*=20 fish). (C) Images of both GPI-linked and cytoplasmic pHluorin2 expressed under the control of the *myosin6b* promoter in hair cells of the inner ear. (D) The extracellular and intracellular environment of inner ear hair cells is acidified in *merovingian* mutants, as measured by a decreased 405/488 ratio in the ratiometric fluorescent pH indicator pHluorin2 (*n*=15–19 fish). (E) Images of both GPI-linked and cytoplasmic pHluorin2 expressed under the control of the *myosin6b* promoter in hair cells of the lateral line. (F) The extracellular, but not intracellular, environment of lateral line hair cells is acidified in *merovingian* mutants, as measured by a decreased 405/488 ratio in the ratiometric fluorescent pH indicator pHluorin2 (*n*=13–16 fish). Values were normalized to the 405/488 ratio of wild-type siblings for each construct. *P<0.05, ***P<0.001 by ANOVA and Bonferroni post-hoc test. Error bars indicate s.d. Scale bars: 10 μm.

For our experiments, we used 405-nm and 488-nm excitation lasers to excite the two peaks of pHluorin2. Given the known properties of pHluorin2, the ratio of 405-nm/488-nm fluorescence intensities should decrease with decreasing pH. pHluorin2 was expressed ubiquitously under the control of the *β-actin* promoter (Kwan et al., 2007). We analyzed muscle cells due to their robust expression of this construct. We used a GPI-linked pHluorin2 (Caras et al., 1987; Miesenbock et al., 1998) to monitor extracellular pH and an untagged cytoplasmic pHluorin2 construct to monitor intracellular pH (Fig. 6A). We found that the 405/488 fluorescence ratio was decreased for both GPI-linked and to a lesser degree for cytoplasmic pHluorin2 in *merovingian* mutants, suggesting that there is both an acidified extracellular and intracellular environment in these animals (Fig. 6B).

To test whether the extracellular environment of hair cells was similarly acidified, we expressed cytoplasmic and GPI-linked pHluorin2 under the control of the hair cell-specific *myosin6b* promoter (Obholzer et al., 2008) (Fig. 6C,E). We found that the GPI-link pHluorin2 construct showed a decreased 405/488 fluorescence ratio in *merovingian* mutants in both lateral line and inner ear hair cells, which is indicative of an acidified extracellular environment of these cells (Fig. 6D,F). Inner ear hair cells, similar to muscle cells, also showed a reduction in the 405/488 fluorescence ratio of cytoplasmic pHluorin2 (Fig. 6D). By contrast, lateral line hair cells showed the same cytoplasmic pHluorin2 405/488 fluorescence ratio in both wild-type siblings and *merovingian* mutants (Fig. 6F). Thus, although the extracellular environment of these cells is acidified in *merovingian* mutants, they are able to maintain a normal intracellular pH.

## DISCUSSION

Exposure to certain therapeutic drugs, particularly aminoglycoside antibiotics and chemotherapeutics, can damage hair cells and cause subsequent hearing loss. However, there is a large amount of variation seen in the degree of hearing loss that patients suffer when taking these drugs (Mulheran et al., 2001; Rybak et al., 2009; Skinner et al., 1990; Xie et al., 2011). This variability is due in part to genetic differences between patients. Although some genes have been identified that alter the susceptibility to drug-induced hearing loss (Hema Bindu and Reddy, 2008; Guan, 2011; Mukherjea and Rybak, 2011; Oldenburg et al., 2008), the picture is far from complete. To identify candidate genes and pathways regulating hair cell susceptibility to ototoxic drugs, we have used the zebrafish lateral line system to screen for mutants involved in aminoglycoside toxicity (Owens et al., 2008). To date, our studies have identified three novel genes involved in ototoxicity: *cc2d2a* (Owens et al., 2008), *slc4a1b* (Hailey et al., 2012) and *gcm2* (this work). Two of these genes, *slc4a1b* and *gcm2*, are involved in pH regulation and both genes cause a decrease in whole body proton extrusion when knocked down in zebrafish (Chang et al., 2009; Lee et al., 2011). We hypothesize that this will cause an internal acidification of the animal and, indeed, our data using pHluorin2 confirm that this is the case in *gcm2* mutants. *gcm2* is involved in parathyroid rather than ionocyte development in humans (Ding et al., 2001; Zajac and Danks, 2008) and therefore is not necessarily involved in human ototoxicity; however, our findings do support a key role for pH regulation in hair cell death that might be conserved across species.

Resistance to toxicant-induced hair cell death in both *gcm2* and *slc4a1b* mutants appears to be due to reduced drug uptake, as both mutants show impaired aminoglycoside and FM1-43 uptake into hair cells (this work; Hailey et al., 2012). Additionally, resistance to neomycin-induced hair cell death is more dramatic than that to cisplatin-induced hair cell death in both mutants (this work; Hailey et al., 2012). In the case of *gcm2*, this difference is consistent with the degree to which uptake is impaired. Reduced FM1-43 uptake along with behavioral abnormalities in *gcm2* mutants suggests that the effect on drug uptake might be due to defects in mechanotransduction. Although the uptake of both aminoglycosides and cisplatin is mechanotransduction-dependent (Alharazneh et al., 2011; Gale et al., 2001; Marcotti et al., 2005; Thomas et al., 2013), the specific mechanisms of their uptake might differ. This idea is consistent with the fact that drugs that protect against aminoglycosides by blocking uptake do not always protect against cisplatin (Vlasits et al., 2012).

In fish, acid excretion occurs primarily at the gills rather than the kidneys (Claiborne et al., 2002). H^+^-ATPase-rich ionocytes have been shown to be important for acid secretion in larval zebrafish (Lin et al., 2006). Because these cells are absent in *merovingian* mutants, we hypothesized that there would be a global acidification of the animal’s internal environment. To confirm that *merovingian* mutants have an acidified internal environment, we used the genetically encoded pH indicator pHluorin2 (Mahon, 2011; Miesenböck et al., 1998). These results show that the extracellular environment of muscle cells as well as inner ear and lateral line hair cells in *merovingian* mutants is acidified, consistent with a whole body acidification. Additionally, the intracellular environment of both muscle and inner ear hair cells are also acidified in *merovingian* mutants, although to a lesser degree than the extracellular environment. By contrast, lateral line hair cells only show an extracellular acidification. As lateral line hair cells are on the surface of the animal it makes sense that they would have additional mechanisms to control their intracellular pH. Indeed, we found an enrichment of H^+^-ATPase staining around the hair cells of the lateral line and this staining was still present in *merovingian* mutants. These data support our hypothesis that *gcm2* functions globally to control whole body pH instead of locally at the hair cells. Additionally, it suggests that the defects we are seeing in *merovingian* mutants are due to changes in extracellular rather than intracellular pH.

Cellular pH regulation has previously been shown to regulate cell death processes, although this regulation is complex (Matsuyama and Reed, 2000). Extracellular acidification influences the response of cancer cells to cisplatin, making cells more susceptible (Atema et al., 1993; Groos et al., 1986; Laurencot et al., 1995; Murakami et al., 2001). However, aberrant cellular pH regulation is also a hallmark of many cancers (Harguindey et al., 2005), which makes it difficult to extend these conclusions to other cell types. Transient application of an acidic solution to the round window potentiated cisplatin ototoxicity in mammals (Tanaka et al., 2003; Tanaka et al., 2004), in contrast to our findings that suggest an acidic environment can partially protect lateral line hair cells from cisplatin. Several differences might account for these different findings. Tanaka and colleagues used transient application of an acidic solution, whereas our mutants are presumably chronically exposed to an acidified environment. Alternatively, mammalian hair cells might use alternative mechanisms of cisplatin uptake that are less sensitive to pH or perturbations in mechanotransduction. It has previously been shown that, unlike in zebrafish, mammalian copper transporters Oct2 and Ctr1 appear to play a role in cisplatin ototoxicity (Ciarimboli et al., 2010; Ding et al., 2011; More et al., 2010; Thomas et al., 2013). It is therefore possible that acidification of the mammalian hair cell environment would not have the same protective effects.

There are multiple possible mechanisms by which acidification of the hair cell environment could lead to defects in hair cell function. Mutations in the H^+^-ATPase subunit *Atp6v0a4* as well as pharmacological manipulations of pH regulatory measures cause dramatic decreases in endocochlear potential (EP) (Ikeda et al., 1987; Kuijpers and Bonting, 1970; Lorente-Cánovas et al., 2013; Norgett et al., 2012; Sterkers et al., 1984; Wangemann et al., 2004). The Na^+^-K^+^-ATPase has been shown to have impaired function at acidic pH (Kuijpers and Bonting, 1969), leading to the hypothesis that inhibition of this pump leads to the decrease in EP seen in an acidified environment (Kuijpers and Bonting, 1970). The cupula of *Xenopus* has been shown to have an elevated endocupular potential and increased K^+^ concentration (Russell and Sellick, 1976), therefore a similar mechanism of action could occur in the lateral line.

Alternatively, altered pH homeostasis might be affecting hair cell function by influencing Ca^2+^ regulation. Fish raised in an acidic environment or with knocked down H^+^-ATPase function show decreased whole body Ca^2+^ levels (Horng et al., 2007; Horng et al., 2009). Mutations in Ca^2+^-modulating proteins are associated with defects in otolith and otoconia formation (Cruz et al., 2009; Hughes et al., 2007; Kozel et al., 1998; Lundberg et al., 2006). Because CaCO_3_ is a major otolith component, a decrease in Ca^2+^ levels could be responsible for the otolith formation defects in *gcm2* mutants. Acidification of the endolymph has also been associated with an increase in endolymphatic Ca^2+^ in the *Pendrin* mutant due to inhibition of the Ca^2+^ channels TRPV5 and TRPV6 (Nakaya et al., 2007; Wangemann et al., 2007). Additionally, acidification of the external environment around hair cells can cause decreased Ca^2+^ entry into hair cells through voltage-gated Ca^2+^ channels (Ikeda et al., 1991; Tan et al., 2001). Proper pH regulation is also probably needed for Ca^2+^ extrusion from hair cell bundles (Hill et al., 2006; Ikeda et al., 1992). It has previously been shown that altered Ca^2+^ levels have dramatic effects on hair cell function and mechanotransduction (Beurg et al., 2010; Ceriani and Mammano, 2012; Ohmori, 1985; Tanaka et al., 1980).

Human patients with distal renal tubular acidosis (dRTA) caused by mutations in subunits of the H^+^-ATPase transporter show sensorineural hearing loss (Batlle and Haque, 2012; Karet et al., 1999; Smith et al., 2000). Patients with dRTA often show hypercalciuria and hypokalemia; however, these K^+^ and Ca^2+^ imbalances are seen in dRTA caused by multiple genetic mutations, including those not associated with sensorineural hearing loss (Batlle et al., 2001; Batlle et al., 2006). Although bicarbonate therapy can help with the acidosis in patients with dRTA, there are no effective therapies to improve hearing impairment (Batlle et al., 2001). The relative ease of manipulating the ionic environment of lateral line hair cells makes the zebrafish a useful model for further studies into the ionic mechanisms behind pH regulation of hair cell function.

## MATERIALS AND METHODS

### Animals

All experiments were performed on 5-day post-fertilization (dpf) *Danio rerio* (zebrafish) larvae, unless otherwise noted. Larvae were obtained by mating adult fish by standard methods (Westerfield, 2000). The *AB wild-type strain was used for these experiments and the *merovingian* (*mero^w40^*) mutant stock was maintained as heterozygotes in the *AB wild-type background. Genetic mapping used the Tübingen strain. All uptake experiments were performed in fish containing the *Tg(pou4f3:gap43-GFP)^256t^* transgene (Xiao et al., 2005); this transgene is referred to here as *brn3c:gfp*. Larvae were raised in embryo media (EM) consisting of 1 mM MgSO_4_, 150 μM KH_2_PO_4_, 42 μM Na_2_HPO_4_, 1 mM CaCl_2_, 500 μM KCl, 15 mM NaCl and 714 μM NaHCO_3_ at pH 7.2. pH was adjusted with NaOH and HCl. Given the 15 mM NaCl present in EM, changes in counterion concentrations during pH adjustments were negligible. The University of Washington Institution Animal Care and Use Committee approved all experiments.

### Otolith measurements

For quantification of otolith size, fish were anesthetized using MS222 and immobilized in 1% low-melting-point agarose on a microscope slide. Fish were imaged on a Zeiss Axioplan 2 microscope using a Spot camera and Spot Advanced Imaging software (version 4.0.6). The posterior otolith was used for size measurements, and area quantification was carried out using ImageJ software (version 1.45s).

### Immunohistochemistry

Zebrafish larvae were fixed in 4% paraformaldehyde in PBS for either 2 hours at room temperature or overnight at 4°C. For parvalbumin staining, fish were washed three times with PBS containing 0.1% Tween 20 (PBST), then incubated for 30 minutes in distilled water, at least 1 hour in antibody block (5% heat-inactivated goat serum in 1× PBS, 0.2% Triton, 1% DMSO, 0.02% sodium azide and 0.2% BSA), and overnight at 4°C in mouse anti-parvalbumin antibody (Millipore, MAB1572) diluted 1:500 in antibody block. Fish were then washed three times in PBST and incubated with fluorescently conjugated secondary antibody (Life Technologies) diluted 1:1000 in antibody block for 4 hours at room temperature, washed three times in PBST and stored in a 50:50 mixture of PBS and glycerol before use.

For vH-ATPase staining, fish were fixed as before, washed three times with PBST, once with 50% MeOH in PBST, once with 100% MeOH, and then stored overnight at −20°C in fresh 100% MeOH. Fish were then washed once with 50% MeOH in PBST, once with PBST, and incubated with antibody block and antibody for the same durations as parvalbumin antibody staining. A rat antibody against the H^+^-ATPase B subunit of dace (*Tribolodon hakonensis*) vh-ATPase, similar to the antibody described in Hirata et al. (Hirata et al., 2003), was used at 1:500 dilution. The vh-ATPase antibody was a gift from Shigehisa Hirose (Department of Biological Sciences, Tokyo Institute of Technology).

### Drug treatment

Animals were exposed to neomycin (Sigma-Aldrich) at the indicated concentrations for 30 minutes in standard EM, washed three times in EM and given 1 hour to recover in EM before being euthanized and fixed. Animals were exposed to cisplatin (Teva, supplied by University of Washington Pharmacy) at the indicated concentrations for 24 hours in standard EM, washed four times in EM and immediately euthanized and fixed. The OP1, M2, IO4, O2, MI2 and MI1 neuromasts (Raible and Kruse, 2000) were counted for all lateral line hair cell number quantifications.

### Neomycin-Texas Red

Neomycin was conjugated to Texas Red-X-succinimidyl ester (Lefevre et al., 1996) in a modified version of the protocols for gentamicin labeling previously described (Sandoval et al., 1998; Steyger et al., 2003). Neomycin sulfate hydrate (Sigma-Aldrich) was used at 115.6 mg/ml final concentration. Neomycin sulfate hydrate solid was resuspended in deionized water up to 50% of the final solution volume, then 0.5 M K_2_CO_3_ at pH 9.0 was added at 17.6% final volume. Texas Red-X-succinimidyl ester (Life Technologies) was dissolved in dimethylformamide at 2.5 mM and was added at 12% final volume. The volume of the mixture was brought to 100% with deionized water and the solution incubated overnight at 4°C to allow the conjugation reaction to go to completion.

### Uptake experiments

For uptake experiments, fish were labeled with 2.25 μM FM1-43FX (Life Technologies) for 1 minute, 50 μM neomycin-TR for 15 minutes, or 25 μM Rhodamine-Universal Labeling System (Rho-Pt, Kreatech Diagnostics; Thomas et al., 2013) for 20 minutes. Fish were exposed to the indicated compound, washed three times and then imaged. To image drug uptake, fish were anesthetized in MS222 and transferred to a Nunc Lab-Tek Chambered Coverglass (Fisher Scientific) where they were immobilized under a nylon mesh and two stainless-steel slice hold-downs (Warner Instruments). One neuromast per fish was imaged, and each neuromast was imaged as a stack of 30 1-μm sections. Image stacks were obtained and analyzed using SlideBook software (version 5.5) running a Marianas spinning disk confocal system (Intelligent Imaging Innovations). Maximum projection images were generated of the entire neuromast stack (for FM1-43 and neomycin-TR labeling), or from nine planes (for Rho-Pt labeling). A mask was drawn around the neuromast based on the *brn3c-gfp* labeling, and the average intensity was calculated. An identical mask was drawn away from the region of the neuromast to calculate the background intensity. Data is shown as neuromast/background intensity.

### Genetic mapping

Heterozygous carriers of the *merovingian* mutation in the *AB strain background were crossed to the Tübingen strain. Hybrid *AB/Tübingen carriers of the *merovingian* mutation were identified by phenotype and intercrossed to generate progeny for linkage marker analysis. Mutant and wild-type fish were selected based on otolith and vestibular phenotypes as well as resistance to 200 μM neomycin. For bulk segregant analysis, two pools of 20 mutants and two pools of 20 wild-type fish were used. Microsatellite markers for each chromosome (Knapik et al., 1998; Shimoda et al., 1999) were amplified by PCR and evaluated for co-segregation with mutant phenotypes. Markers co-segregating with the *merovingian* allele were further evaluated with individual DNA from 294 mutant fish and 32 wild-type fish. Initial mapping localized the mutation between Z-markers Z23011 and Z24856. To narrow the region further, candidate SSR marker primer pairs for this work were generated using the Zebrafish Genome SSR search website (Massachusetts General Hospital, Charlestown, MA 02129; http://danio.mgh.harvard.edu/chrMarkers/zfssr.html). To sequence candidate genes, RNA was isolated from pools of 20 wild-type sibling or mutant embryos using TRIzol Reagent (Life Technologies). cDNA was prepared using SuperScript III Reverse Transcriptase (Life Technologies). Genes were amplified by PCR from cDNA and then sent to Eurofins MWG Operon for sequencing.

### *gcm2* morpholino oligonucleotide

For knock-down experiments, we used a previously described *gcm2* antisense morpholino oligonucleotide (Hanaoka et al., 2004) with the sequence 5′-AAACTGATCTGAGGATTTGGACATG-3′ (Gene Tools, LLC). The MO (in 0.1% Phenol Red) was injected into the yolk of 1-cell stage embryos at 10 ng/embryo using previously described techniques (Nasevicius and Ekker, 2000). For a mock injection negative control, 0.1% Phenol Red was injected at comparable volumes as the MO injections.

### pHluorin2

*pHluorin2* DNA was obtained from Matthew Mahon (Massachusetts General Hospital, Harvard Medical School). Constructs were generated to express *pHluorin2* (Mahon, 2011) under the control of the *β-actin* and *myosin6b* (*myo6b*) promoter in a Tol2 transposon backbone (Kwan et al., 2007) using standard Gateway cloning mechanisms (Walhout et al., 2000). The GPI targeting sequence of folate receptor alpha (Lacey et al., 1989) was fused to pHluorin2 to generate GPI-pHluorin2. DNA constructs were injected into single-cell embryos at 200 pg along with 40 ng of transposase mRNA. Transiently injected fish expressing pHluorin2 under the control of the *β-actin* promoter were used for quantification of muscle cells. For hair cell experiments, injected fish were grown to adulthood and screened for germline incorporation of the transgene. Two stable lines were generated, *Tg(myo6b:pHluorin2)^w134^* and *Tg(myo6b:pHluorin2gpi)^w135^*, and used in all hair cell pHluorin experiments. Microscope and immobilization techniques used for uptake experiments (see above) were used for pHluorin imaging. For muscle cells, a 20-section stack of 1-μm sections was collected containing trunk muscle cells. For hair cells, a 30-section stack of 1-μm sections was collected from either the anterior crista or anterior lateral line. pHluorin2 fluorescence was acquired using both the 405 and 488 excitation lasers and a 535/30 emission filter. Single planes were used for image analysis. For hair cell data, the cell body was used for measurements. Background correction was carried out in SlideBook software. Fluorescence elicited by the 405 and 488 excitations was measured and then ratioed. One cell was analyzed per animal.

### Statistical analyses

All statistics were calculated using the GraphPad Prism software (GraphPad, version 4.0). All data are represented as means and standard deviations. *P*-values are based on ANOVA and Bonferroni post-hoc tests or the Student’s *t*-test.

## Supplementary Material

Supplementary Material
