# Supplementary material for: The zebrafish merovingian mutant reveals a role for pH regulation in hair cell toxicity and function
Source: Dis Model Mech. 2014 Jul;7(7):847–56. doi: 10.1242/dmm.016576 (PMC4073274; doi:10.1242/dmm.016576)
Supplement: Supplementary Material [file supp_7_7_847__index.html]

The zebrafish merovingian mutant reveals a role for pH regulation in hair cell toxicity and function — Supplementary Material 

# The zebrafish *merovingian* mutant reveals a role for pH regulation in hair cell toxicity and function

## DMM016576 Supplementary Material

**Files in this Data Supplement:**

- **Supplementary Material**
