# Supplementary material for: The zebrafish merovingian mutant reveals a role for pH regulation in hair cell toxicity and function
Source: Dis Model Mech. 2014 Jul;7(7):847–56. doi: 10.1242/dmm.016576 (PMC4073274; doi:10.1242/dmm.016576)
Supplement: Supplementary Material [file supp_7.7.847_DMM016576.pdf]

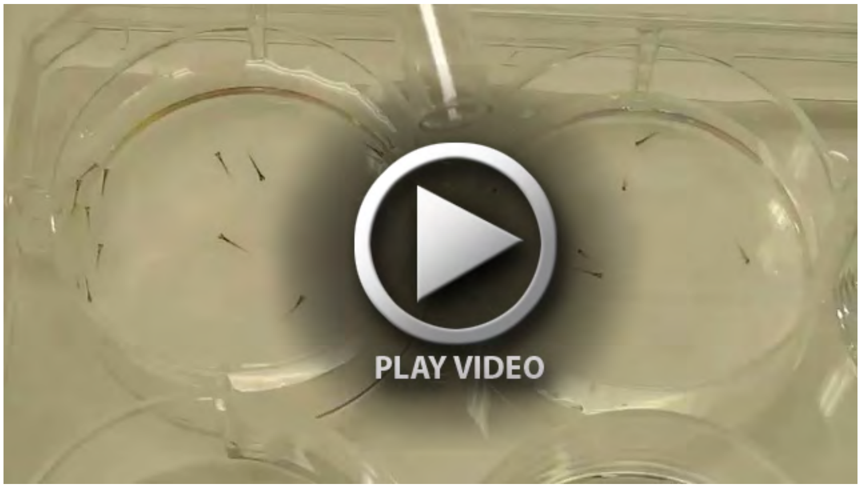

Movie 1. *merovingian* mutants fail to respond to acoustic/vibrational stimuli. Wild type fish show a startle response after vibrations are generated by taping on the dish (left). This response is absent in *merovingian* mutants (right). *merovingian* mutants also show a failure to maintain an upright body position.

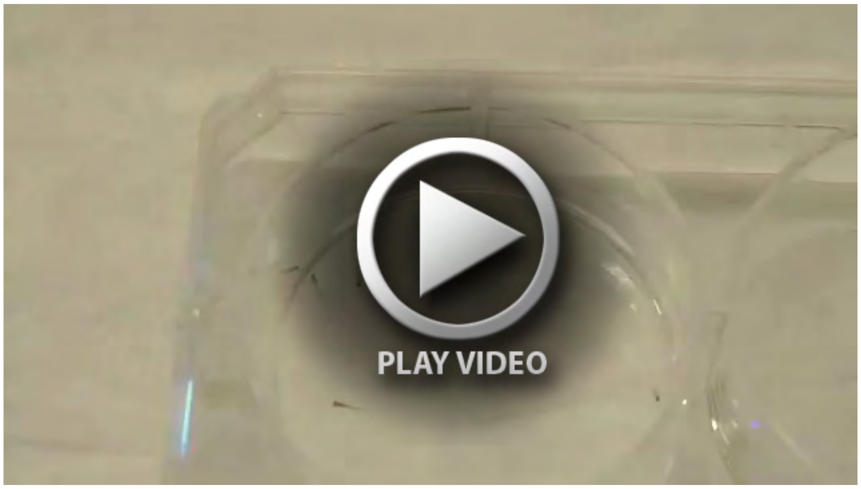

Movie 2. *merovingian* mutants show circling behavior. Video of *merovingian* mutants one of which is showing circling behavior.
